# Genetically Supported Causality between Brain Structural Connectome and Sleep Duration in Children: A Two-Sample Mendelian Randomization Study

**DOI:** 10.1523/ENEURO.0267-24.2024

**Published:** 2024-12-12

**Authors:** Ruijie Zhang, Liyan Luo, Lu Zhang, Xinao Lin, Chuyan Wu, Feng Jiang, Jimei Wang

**Affiliations:** ^1^Department of Neonatology, Obstetrics and Gynecology Hospital of Fudan University, Shanghai 200011, China; ^2^Department of Neonatology, Dali Bai Autonomous Prefecture Maternal and Child Health Care Hospital, Dali 671000, China; ^3^Department of Rehabilitation Medicine, The First Affiliated Hospital of Nanjing Medical University, Nanjing 210029, China

**Keywords:** brain networks, children, Mendelian randomization, sleep duration, structural connectivity

## Abstract

Certain structural brain connections have been confirmed to influence sleep duration in children. However, the causal relationships between all brain regions and children's sleep duration remain unclear. A two-sample Mendelian randomization analysis was conducted using data from genome-wide association studies (GWAS) to examine the relationships between 206 structural connections and sleep duration in children. Sensitivity analyses were employed to validate the findings and assess the robustness of the causal inferences. Stronger connectivity from the left hemisphere (LH) control network to the accumbens (*β* = −0.15; 95% CI = [−0.30, −2.88 × 10^−3^]; *p* = 0.05) and from the LH somatomotor network to the LH default network (*β* = −0.18; 95% CI = [−0.34, −0.03]; *p* = 0.02) in white-matter structural connectivity (SC) were associated with shorter sleep durations. Conversely, increased white-matter SC from the LH dorsal attention network to the thalamus (*β* = 0.14; 95% CI = [8.45 × 10^−4^, 0.27]; *p* = 0.05), from the right hemisphere (RH) control network to the thalamus (*β* = 0.10; 95% CI = [0.01, 0.19]; *p* = 0.03), from the RH default network to the thalamus (*β* = 0.08; 95% CI = [4.53 × 10^−3^, 0.16]; *p* = 0.04), from the RH limbic network to the thalamus (*β* = 0.15; 95% CI = [0.05, 0.26]; *p* = 3.77 × 10^−3^), and from the RH somatomotor network to the thalamus (*β* = 0.20; 95% CI = [0.07, 0.32]; *p* = 1.63 × 10^−3^) correlated with longer sleep durations in children. Two-sample Mendelian randomization provides novel insights into the relationships between brain regions and sleep duration in children. Our findings demonstrate a causal relationship between specific brain areas and sleep duration.

## Significance Statement

This study explores neurobiological mechanisms underlying sleep regulation in children, revealing causal links between brain structural connectivity and sleep duration through Mendelian randomization. Using genetic variants as instrumental variables, 206 structural connections were analyzed, identifying pathways involving networks such as the dorsal attention, somatomotor, and default mode networks. These findings highlight the role of neural architecture in shaping sleep patterns, advancing understanding of developmental sleep regulation. The results have important implications for pediatric health, offering insights for targeted interventions and biomarkers to enhance sleep quality, ultimately supporting cognitive and behavioral development.

## Introduction

Sleep is essential for physical and mental health during childhood development (Astill et al., 2012). Despite recommendations from the American Academy of Sleep Medicine for children aged 6–18 to sleep between 8 and 12 h per night (Paruthi et al., 2016), >90% of school-aged children do not meet these guidelines, with sleep duration decreasing as they approach adolescence (Gradisar et al., 2011). Adolescent sleep patterns are influenced by genetic factors; for instance, genetics account for ∼48% of the variance in sleep spindles (Markovic et al., 2020). Systematic reviews and genetic studies employing genome-wide association studies (GWAS) corroborate that sleep duration in children is also genetically influenced (Marinelli et al., 2016; Kocevska et al., 2021).

Brain structural connectivity (SC) has demonstrated significant genetic correlations with various neuropsychiatric and cognitive traits (Wainberg et al., 2024). However, the influence of SC on sleep duration in children has received less attention. Existing research indicates a relationship between brain networks and children's sleep patterns. For example, connections within the default mode network (DMN), which are prominent during periods of mental rest, are consistently associated with sleep and other neuroregulatory behaviors (Tashjian et al., 2017). Total sleep deprivation has been shown to disrupt interactions between the DMN and core cognitive networks, including the dorsal attention network (DAN), frontoparietal network (FPN), and salience network (SN; Fox et al., 2005; Lei et al., 2015; Dai et al., 2020). However, these studies have focused only on specific brain regions and are often subject to confounding factors. To date, a comprehensive examination of the connection between brain networks and children's sleep duration from a developmental perspective is lacking.

In this study, we aim to address the gap in understanding the causal links between brain SC and sleep duration in children using Mendelian randomization (MR). MR leverages genetic variants, such as single nucleotide polymorphisms (SNPs), to infer causal relationships by minimizing the impact of confounding factors (Gupta et al., 2017; Davies et al., 2018; Yao et al., 2022). Since SNPs are randomly assigned, they are less affected by environmental or disease-related influences, providing a more precise understanding compared with traditional observational studies. Through MR, we explore how specific brain networks might influence sleep duration in children. While our findings offer insights into potential regulatory roles of these networks, further studies are needed to validate these results and clarify the underlying mechanisms.

## Materials and Methods

### Ethics statement

All the GWAS data utilized in this research were sourced from publicly accessible databases, and no original data were collected for this study. Each of the studies included had received approval from their respective institutional ethics review committees. Additionally, informed consent for both participation and publication was obtained from all participants involved.

### Study design

**Figure 1.**
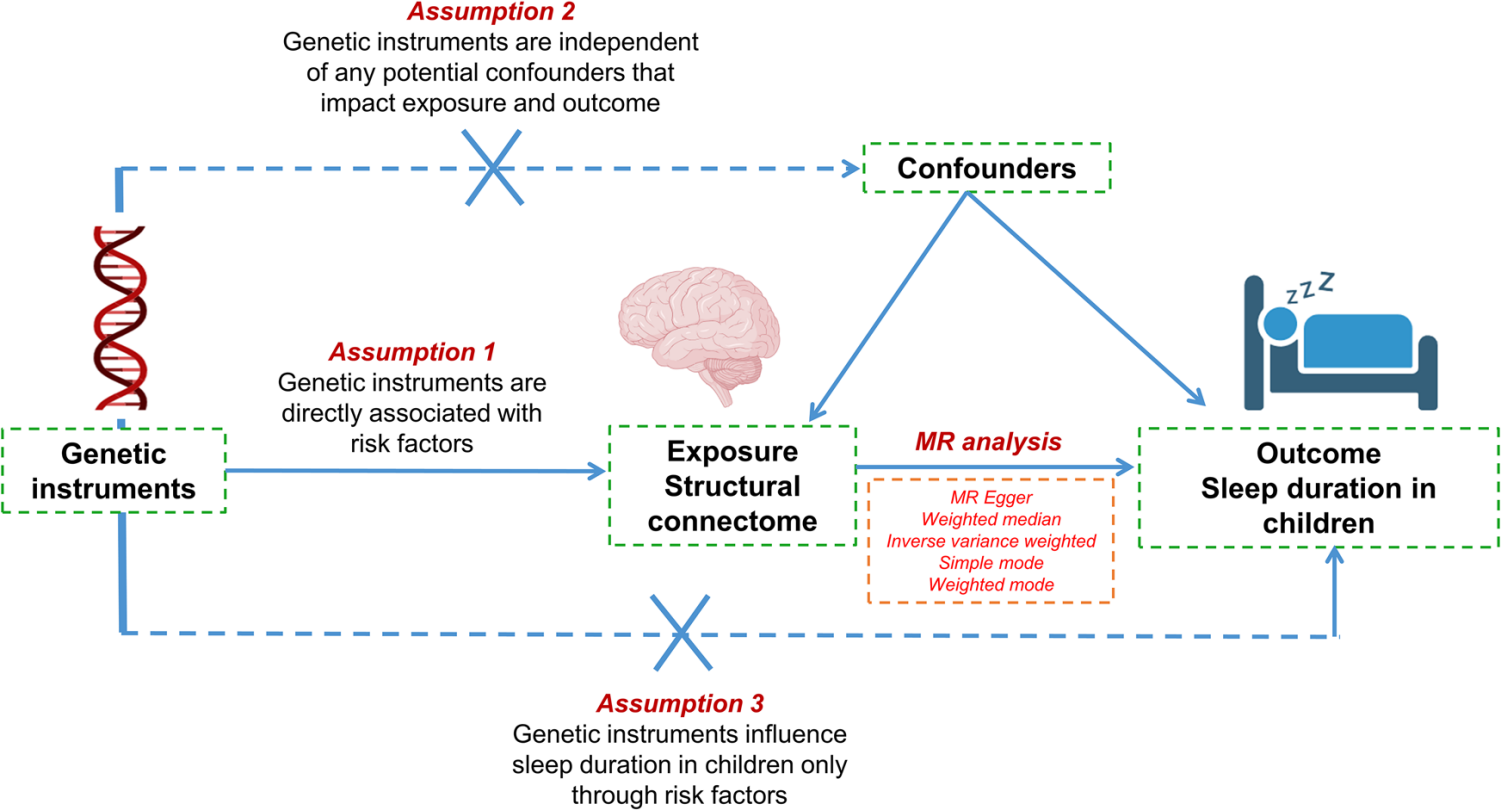
Schematic overview of the study design for investigating the causal relationships between structural connectome measures and sleep duration in children using MR. The diagram outlines the three main assumptions of our Mendelian randomization study: (1) Genetic instruments are strongly associated with the exposure (structural connectome); (2) the genetic instruments are not associated with any confounders that could influence both the exposure and the outcome (sleep duration); and (3) the influence of the genetic instruments on the outcome occurs exclusively through the exposure, with no direct effects on the outcome as independent factors.

A schematic overview of the study design is depicted in Figure 1. We conducted a Mendelian randomization (MR) investigation utilizing data from two publicly accessible genome-wide association studies (GWAS) to acquire summary statistics. The three assumptions underlying our experiment are illustrated in Figure 1: (1) Genetic instruments, serving as instrumental variables (IVs), are strongly associated with the exposure variable, namely, the structural connectome. (2) Genetic instruments are not associated with any potential confounders that could affect both the exposure and the outcome (sleep duration in children). (3) The influence of genetic instruments on the outcome is mediated exclusively through the exposure, with no direct effects on the outcome independent of the exposure.

All studies involved in the GWAS had previously been approved by the appropriate review boards; therefore, no further ethical approval or participant consent was necessary for this analysis.

### Source of GWAS data

Details of the studies and datasets used for analyses are shown in Table 1. Sleep duration in children was reported by parents, including nap times, and recorded in hours. Parents were asked the open-ended question, “How many hours does your child sleep per day, including nap time?” The cohort included children aged 2–14 years of European ancestry, deliberately excluding those under 2 years of age to avoid the sleep development stage and those over 14 to circumvent the adolescent developmental period (Marinelli et al., 2016). The discovery phase used data from five cohorts in four countries (United Kingdom, Spain, Netherlands, Germany), and the replication phase added two cohorts from two additional countries (Netherlands, Finland), making a total of six countries. The GWAS summary statistics can be accessed and downloaded from the Sleep Disorder Knowledge Portal (https://sleep.hugeamp.org/downloads.html).

**Table 1.** Details of studies and datasets used for analyses

Exposure/outcome	Consortium or cohort study	Participants	PMID
Structural connectome	Michael et al.'s study	26,333 individuals of European ancestry	38438384
Sleep duration in children	Marinelli et al.'s study	10,554 cases of European ancestry	27568811

The network connectivity measures included a total of 206 phenotypic traits (Wainberg et al., 2024). Initially, three hemisphere-level connectivities were assessed: left intrahemisphere, right intrahemisphere, and interhemisphere connectivity. Subsequently, 105 network-level connectivity measures were analyzed, comprising 14 within-network and 91 between-network connectivity measures. These were derived from 14 large-scale brain networks across the left and right hemispheres, known as the “Yeo 7” networks (Yeo et al., 2011), which include seven networks per hemisphere arranged in various combinations, including self-combinations. The Yeo 7 networks consist of visual (“Vis”), somatomotor (“SomMot”), dorsal attention (“DorsAttn”), salience/ventral attention (“SalVentAttn”), limbic, control (“Cont”), and default mode (“Default”). Lastly, 98 cortical-to-subcortical connectivity measures were evaluated, involving combinations of the 14 hemisphere-specific networks with seven subcortical structures. These subcortical structures include the thalamus, caudate, putamen, pallidum, hippocampus, amygdala, and accumbens. Genome-wide summary statistics can be found at the European Bioinformatics Institute GWAS Catalog (https://www.ebi.ac.uk/gwas) under accession numbers GCST90302648 through GCST90302853.

### Two-sample MR analysis

To ensure accurate and reliable outcomes in studying the relationship between SC and sleep duration in children, several quality control steps were undertaken to select the most appropriate IVs. Firstly, to obtain a sufficient number of SNPs for subsequent sensitivity analyses, we set a threshold of *p* < 1 × 10^−6^ for SNP selection, following previous MR studies on SC measures. Secondly, any SNPs in linkage disequilibrium were excluded if they had an *R*^2^ value greater than 0.001 within a clumping window of 10,000 kb. Thirdly, palindromic SNPs, such as those with A/T or G/C alleles, were excluded to prevent strand ambiguity. Finally, the strength of the IVs was assessed using *F* statistics; an *F* statistic greater than 10 suggests that the likelihood of bias from weak instruments is minimal.

We utilized the two-sample MR approach, employing the TwoSampleMR (version 0.5.11) package (Hemani et al., 2017, 2018). Our principal method for MR analysis was the inverse variance weighting (IVW) method. This meta-analytic approach combines Wald estimates from each SNP to compute an overall effect estimate of the exposure on the outcome (Burgess et al., 2013). This method operates under the assumption that all included variants are valid instruments or that any horizontal pleiotropy is balanced, thus not violating the fundamental assumptions of MR.

We supplemented our primary analysis with several auxiliary methods to further assess causality. The MR-Egger method (Bowden et al., 2015) uses weighted linear regression to address potential biases due to directional pleiotropy by analyzing the relationship between the SNP–exposure and SNP–outcome associations. It offers reliable causal estimates even when all genetic variants may be invalid instruments under the InSIDE (Instrument Strength Independent of Direct Effect) assumption, albeit with less efficiency compared with IVW and median-based methods. The weighted median approach (Bowden et al., 2016) integrates data from multiple genetic variants to produce a single causal estimate and provides consistent estimates even if up to 50% of the instruments are invalid. Mode-based methods (Hartwig et al., 2017) classify SNPs into clusters based on similarity in causal effects, estimating the causal effect from the most frequent value (mode). This approach minimizes bias and reduces type I errors, particularly when the SNPs in the largest cluster are valid instruments. To enhance the robustness of our results, we conducted further verification using Bayesian Weighted Mendelian Randomization (BWMR). BWMR is an advanced Mendelian randomization method that utilizes a Bayesian framework to model genetic associations with both the exposure and the outcome (Zuber et al., 2020). This approach integrates prior distributions and accommodates uncertainties in estimates, enabling more robust causal inference particularly when dealing with weak instruments or complex pleiotropic structures. The MR results obtained from the BWMR analysis demonstrate consistency with our initial findings and confirm the robustness of our results.

### Sensitivity analysis

To enhance the robustness of our findings, we conducted several sensitivity analyses. Firstly, we evaluated the heterogeneity of SNP effects using Cochran's *Q* statistic (Bowden et al., 2018), as heterogeneity could bias the IVW estimates. A *p* value greater than 0.05 for this test suggested minimal heterogeneity impact. Where heterogeneity was detected, we employed the random-effects model of IVW to assess the causal effects more appropriately. Secondly, to address the risk of horizontal pleiotropy, which could violate the exclusion restriction assumption of MR (that IVs should not influence the outcome except through the exposure; Angrist et al., 1996; Didelez and Sheehan, 2007), we analyzed the MR-Egger regression intercept. An intercept not significantly different from zero (*p* > 0.05) indicated an absence of directional pleiotropy (Burgess and Thompson, 2017). Lastly, we implemented a leave-one-out analysis to ascertain the robustness of the causal effect estimates. This approach involves sequentially removing each IV and recalculating the IVW estimate using the remaining IVs, thus assessing the influence of individual variants on the overall causal estimate. These methods collectively ensure our analysis is both thorough and reliable in detecting true causal relationships.

### Data and code accessibility statement

Data and code can be made available on written reasonable request to the corresponding authors.

## Results

### Selection of IVs

Initially, 206 SC measures and 3,056 SNPs were extracted under a locus-wide significance threshold of *p* < 5 × 10^−6^. These 206 SC measures include three hemisphere-level connectivity measures (70 SNPs), 14 within-network connectivity measures (171 SNPs), 91 between-network connectivity measures (1,395 SNPs), and 98 cortical-to-subcortical connectivity measures (1,410 SNPs).

### MR results with five statistical methods

**Figure 2.**
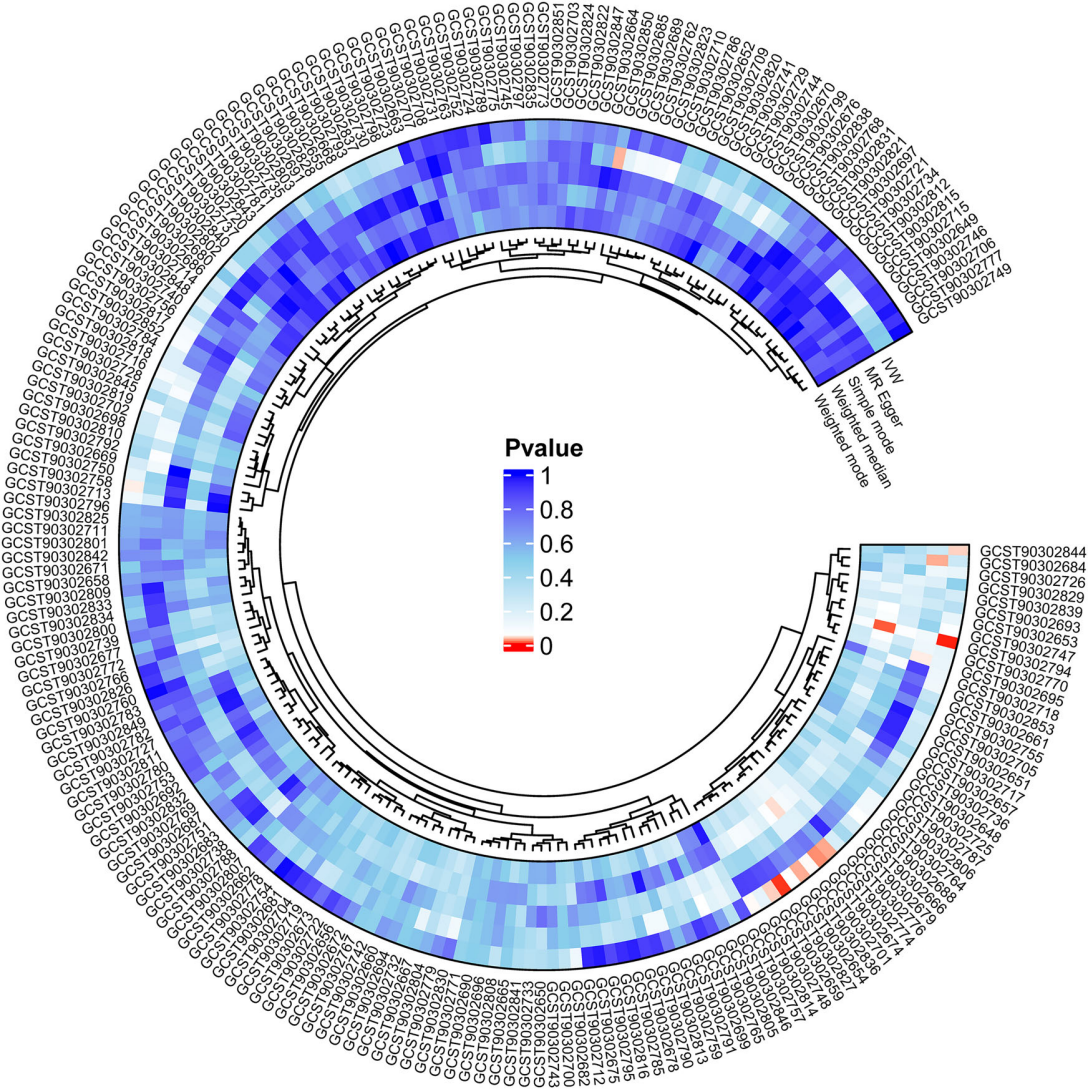
Circos image of the association of 206 SC measures with sleep duration in children. Moving from the inner circle to the outer circle, five statistical techniques are depicted. Red indicates a *p* value of <0.05.

We explored the connections between SC measures and sleep duration in children using a two-sample MR approach. Significant associations were identified between sleep duration and the SC of the LH control network to the accumbens, LH dorsal attention network to the thalamus, LH somatomotor network to the LH default network, RH control network to the thalamus, RH default network to the thalamus, RH limbic network to the thalamus, and RH somatomotor network to the thalamus white matter (*p* < 0.05; Fig. 2).

Table 2 presents findings from the two-sample MR analysis, showing associations between seven SC measures and sleep duration in children. It was observed that higher densities in the white-matter SC from the LH control network to the accumbens [inverse variance weighted (IVW): *β* = −0.15; 95% CI = [−0.30, −2.88 × 10^−3^]; *p* = 0.05] and the LH somatomotor network to the LH default network (IVW: *β* = −0.18; 95% CI = [−0.34, −0.03]; *p* = 0.02) are associated with shorter sleep durations. Conversely, increased densities in the white-matter SC from the LH dorsal attention network to the thalamus (IVW: *β* = 0.14; 95% CI = [8.45 × 10^−4^, 0.27]; *p* = 0.05), the RH control network to the thalamus (IVW: *β* = 0.10; 95% CI = [0.01, 0.19]; *p* = 0.03), the RH default network to the thalamus (IVW: *β* = 0.08; 95% CI = [4.53 × 10^−3^, 0.16]; *p* = 0.04), the RH limbic network to the thalamus (IVW: *β* = 0.15; 95% CI = [0.05, 0.26]; *p* = 3.77 × 10^−3^), and the RH somatomotor network to the thalamus (IVW: *β* = 0.20; 95% CI = [0.07, 0.32]; *p* = 1.63 × 10^−3^) correlate with longer sleep durations in children.

**Table 2.** Two-sample MR analysis on the association between connectivity measures and sleep duration in children

Connectivity measures	nsnp	Method	*β* (95% CI)	SE	*p*
LH Cont network to accumbens white-matter SC	11	IVW	−0.15 (−0.30, −2.88 × 10^−3^)	0.08	0.05
		MR-Egger	−0.19 (−0.63, 0.24)	0.22	0.41
		Simple mode	−0.02 (−0.38, 0.33)	0.18	0.9
		Weighted median	−0.07 (−0.27, 0.13)	0.10	0.51
		Weighted mode	−0.00333 (−0.32, 0.32)	0.16	0.98
LH DorsAttn network to thalamus white-matter SC	17	IVW	0.14 (8.45 × 10^−4^, 0.27)	0.07	0.05
		MR-Egger	0.07 (−0.30, 0.44)	0.19	0.72
		Simple mode	0.21 (−0.08, 0.50)	0.15	0.17
		Weighted median	0.17 (−0.01, 0.34)	0.09	0.06
		Weighted mode	0.18 (−0.06, 0.42)	0.12	0.16
LH SomMot network to LH Default network white-matter SC	10	IVW	−0.18 (−0.34, −0.03)	0.08	0.02
		MR-Egger	−0.10 (−0.73, 0.52)	0.32	0.75
		Simple mode	−0.25 (−0.57, 0.07)	0.17	0.17
		Weighted median	−0.21 (−0.4, −0.01)	0.10	0.04
		Weighted mode	−0.26 (−0.57, 0.04)	0.16	0.13
RH Cont network to thalamus white-matter SC	27	IVW	0.10 (0.01, 0.19)	0.05	0.03
		MR-Egger	0.06 (−0.21, 0.32)	0.14	0.67
		Simple mode	0.09 (−0.12, 0.29)	0.11	0.41
		Weighted median	0.08 (−0.04, 0.19)	0.06	0.18
		Weighted mode	0.08 (−0.04, 0.21)	0.06	0.21
RH Default network to thalamus white-matter SC	27	IVW	0.08 (4.53 × 10^−3^, 0.16)	0.04	0.04
		MR-Egger	0.14 (−0.09, 0.37)	0.12	0.26
		Simple mode	0.11 (−0.11, 0.32)	0.11	0.34
		Weighted median	0.04 (−0.08, 0.16)	0.06	0.51
		Weighted mode	0.06 (−0.08, 0.20)	0.07	0.37
RH Lim network to thalamus white-matter SC	20	IVW	0.15 (0.05, 0.26)	0.05	3.77 × 10^−3^
		MR-Egger	0.03 (−0.28, 0.34)	0.16	0.85
		Simple mode	0.12 (−0.12, 0.35)	0.12	0.34
		Weighted median	0.13 (−0.02, 0.28)	0.08	0.1
		Weighted mode	0.11 (−0.07, 0.28)	0.09	0.26
RH SomMot network to thalamus white-matter SC	14	IVW	0.20 (0.07, 0.32)	0.06	1.63 × 10^−3^
		MR-Egger	0.41 (−0.04, 0.87)	0.23	0.10
		Simple mode	0.26 (0.01, 0.52)	0.13	0.07
		Weighted median	0.22 (0.05, 0.39)	0.09	0.01
		Weighted mode	0.25 (−0.01, 0.51)	0.13	0.08

LH, left-hemisphere; RH, right-hemisphere; Cont, control; DorsAttn, dorsal attention; SomMot, somatomotor; Default, default mode; Lim, limbic; SC, structural connectivity; IVW, inverse-variance weighted; nsnp, number of SNP; SE, standard error; CI, confidence interval.

Results calculated using the IVW method suggest that each standard deviation increase in the white-matter SC from the LH somatomotor network to the LH default network results in a reduction of sleep duration by 0.18 h. Similarly, each standard deviation increase in the white-matter SC from the RH somatomotor network to the thalamus is associated with an increase in sleep duration by 0.20 h in children.

**Figure 3.**
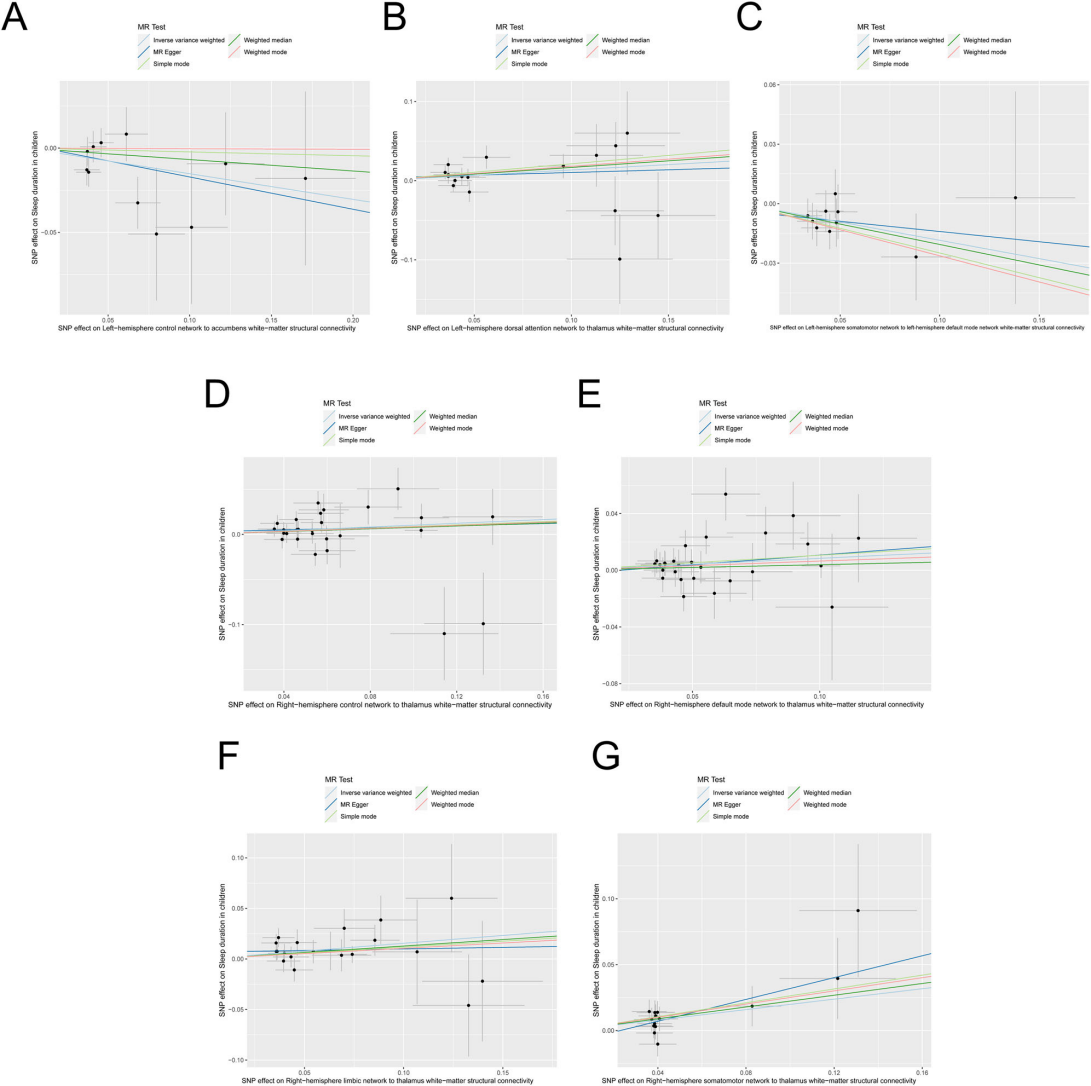
Scatterplot illustrating the two-sample MR results for the effects of the SC of LH Cont network to accumbens (***A***), LH DorsAttn network to thalamus (***B***), LH SomMot network to LH Default network (***C***), RH Cont network to thalamus (***D***), RH Default network to thalamus (***E***), RH Lim network to thalamus (***F***), and RH SomMot network to thalamus (***G***) white-matter on sleep duration in children. The Slop of various colorful lines represent the estimated MR effect derived from different MR methods.

Trend relationships between these seven significant brain structural connections and children's sleep duration are displayed using scatterplots in Figure 3. Each plot utilizes different colors to represent the various MR analysis methods.

### Assessment of assumptions

**Figure 4.**
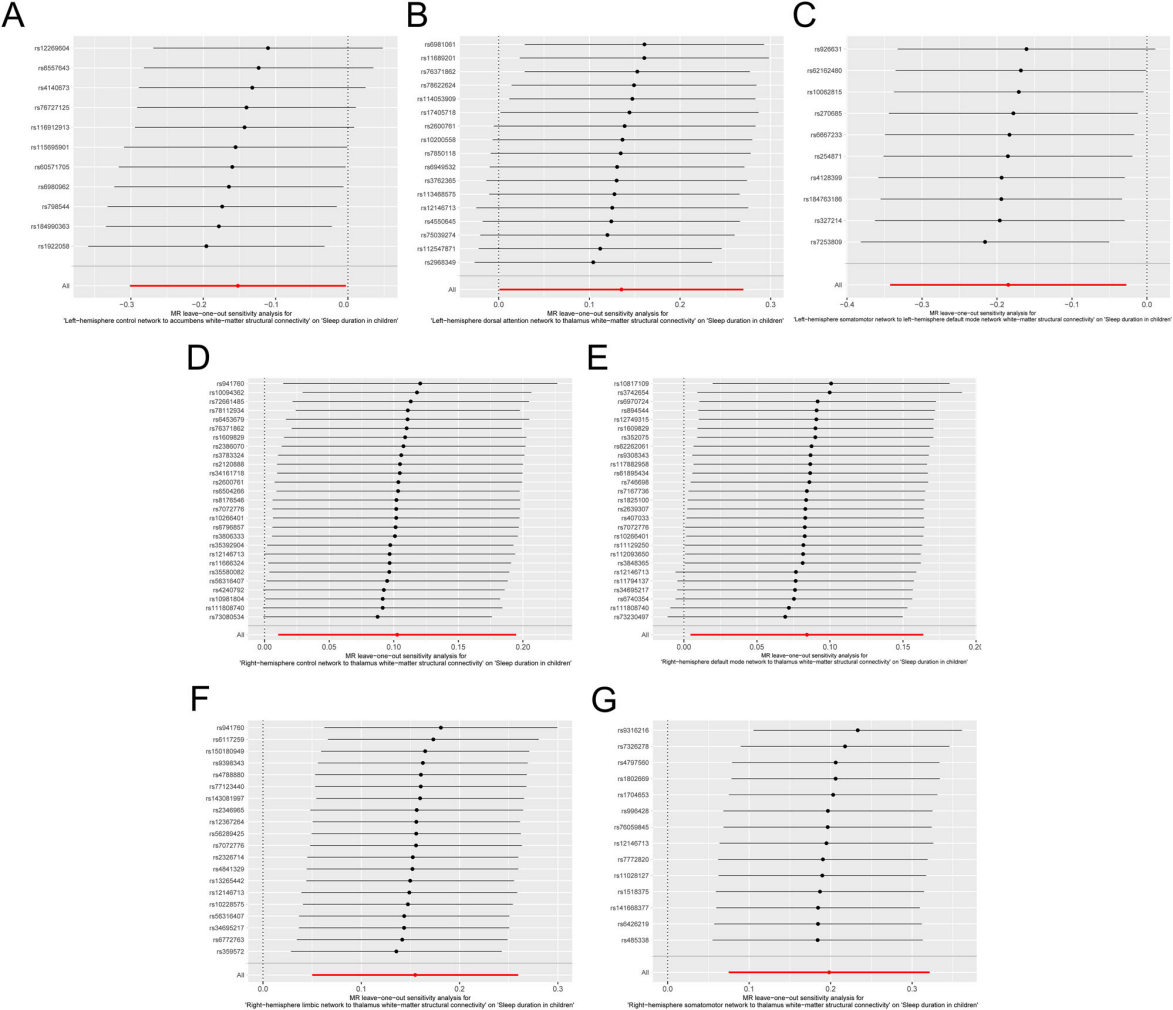
Leave-one-out plots for causal effects for the SC of LH Cont network to accumbens (***A***), LH DorsAttn network to thalamus (***B***), LH SomMot network to LH Default network (***C***), RH Cont network to thalamus (***D***), RH Default network to thalamus (***E***), RH Lim network to thalamus (***F***), and RH SomMot network to thalamus (***G***) white-matter on sleep duration in children on sleep duration in children. Each line indicates the effect of an IV.

Cochran's *Q* test for the IVW findings demonstrated a lack of significant statistical heterogeneity across the independent variables. Additionally, the MR-Egger regression intercepts suggested no significant directional horizontal pleiotropy. The robustness of these findings is further supported by the leave-one-out sensitivity analysis, as shown in Figure 4, confirming that no single IV disproportionately influenced the overall causal assessment.

## Discussion

In this study, we identified that out of 206 brain SC indices, seven showed a causal relationship with children's sleep duration. Specifically, enhancements in the white-matter SC from the LH control network to the accumbens and from the LH somatomotor network to the LH default network were associated with reduced sleep duration. Conversely, increases in the white-matter SC involving the LH dorsal attention network to the thalamus, RH control network to the thalamus, RH default network to the thalamus, RH limbic network to the thalamus, and RH somatomotor network to the thalamus were linked to longer sleep durations.

The SC between the LH control network and accumbens is negatively correlated with children's sleep duration. Within this context, the control network refers to the executive control networks (ECNs), which serve as control systems governing task selection and behavioral guidance, utilizing information from various brain networks to inform individual choices (Koechlin, 2007). Furthermore, the nucleus accumbens (NAcc) is central to goal-oriented and reward-seeking behaviors (Salgado and Kaplitt, 2015) and is associated with anxiety, depression, and addictive behaviors (Lee et al., 2019). The NAcc may serve as an effective target for specific pathologies related to reward-based or pain-related behaviors that exhibit risky characteristics (Yan et al., 2023). Research indicates that enhanced connectivity between reward areas—such as the bilateral anterior cingulate cortex, right posterior orbitofrontal cortex, and ventral striatum—and the LH ECNs is associated with improved executive functioning (Jenkins et al., 2021).

Enhanced SC between the LH somatomotor network (SMN) and LH default network (DMN) is associated with decreased sleep duration in children. The SMN, also known as the sensorimotor network, comprises the precentral gyrus, which governs motor control, and the postcentral gyrus, which processes somatic sensations. The DMN is characterized by higher activity levels during rest and sleep compared with periods of task engagement (Buckner et al., 2009). Studies in adults have identified enhanced connectivity between the SMN and DMN as a marker of early functional pathologies in Huntington's disease (Sánchez-Castañeda et al., 2017). Research has also shown reduced SC in regions such as the left somatomotor area, ventral and dorsal attention networks (SAN), and DMN among patients with insomnia compared with healthy controls (Rostampour et al., 2023). In children, patients with attention-deficit/hyperactivity disorder (ADHD) exhibit lower spontaneous neural activity in the DMN and SMN (Bu et al., 2020). Furthermore, increased connectivity between these networks correlates with more severe hyperactive behaviors. This may help explain why enhanced SMN→DMN connectivity is associated with reduced sleep duration in children.

The SC between the LH dorsal attention network (DAN) and thalamus white matter exhibits a positive correlation with sleep duration in children. The DAN is crucial for maintaining external visuospatial attention, monitoring, and processing visual stimuli within the environment, such as noting specific landmarks or objects during navigation or map reading (Dixon et al., 2017). This network also plays a role in the flow of information from higher-level to lower-level brain areas, facilitating goal-directed behaviors and attentional control (Farrant and Uddin, 2015), enabling individuals to prioritize essential information. The functional connectivity between the thalamus and DAN tends to strengthen with age and is associated with enhanced cognitive abilities, including processing speed, selective attention, and cognitive flexibility (Steiner et al., 2020). However, in children, research has predominantly focused on the functional connectivity between the DMN and DAN. Studies indicate that in children aged 11–13, increased wakefulness after sleep onset is linked with reduced connectivity between the DMN and DAN, particularly in those with shorter sleep duration, with these changes being most pronounced (Hehr et al., 2023). Furthermore, a study of children aged 9–10 revealed that stronger connectivity between the DMN and DAN predicts poorer mental health and sleep outcomes 1 year later (Yang et al., 2022).

The SC between the RH default network and thalamus, RH limbic network and thalamus, and RH somatomotor network and thalamus white matter exhibits a positive correlation with sleep duration in children. The RH default network to thalamus white-matter connectivity is diminished in individuals with mild cognitive impairment (Alderson et al., 2017). Infants born to mothers with prenatal depression demonstrate reduced local clustering and efficiency within the DMN. Furthermore, infant sleep duration positively correlates with overall DMN efficiency (Donnici et al., 2023). Among adolescents, stronger functional connectivity within the DMN during sleep is associated with increased impulsivity under stress, particularly in the context of sleep deprivation (Zhang et al., 2023). The limbic network, responsible for a variety of emotions and long-term memory, projects from the hippocampal region through the fornix to the mammillary bodies and subsequently connects to the thalamus via additional neural pathways (Deasy et al., 2000). Pathological disruptions are observed in the limbic–cortical–striatal–pallidal–thalamic network among patients with severe depression (Zhang et al., 2022). Furthermore, the limbic system network appears to exert a protective effect on sleep, with increased sleep duration associated with enhanced clustering coefficients and local efficiency within the infant limbic system (Donnici et al., 2023). Enhanced SC between the SMN and the thalamus is observed in patients with chronic low back pain and in those with chronic and early-stage psychiatric disorders (Woodward and Heckers, 2016; Zhu et al., 2024). In children, functional connectivity between the ventral region of the thalamus and the facial somatomotor (SMF) network is stronger compared with adults; however, this connectivity begins to diminish starting from the age of 7 (D’Andrea et al., 2023).

Research on the DMN reveals that its maturation during childhood notably involves an increase in connectivity strength and integration, particularly between its anterior and posterior components. These developmental changes in the DMN are crucial for facilitating higher-order cognitive processes and maintaining sleep regulation. Khundrakpam et al. specifically highlight the progressive integration of the DMN with cognitive control networks, which mirrors the development of self-regulation capabilities and sleep maturity during adolescence (Khundrakpam et al., 2016). In our study, alterations in the DMN, particularly the enhanced connectivity from the LH control network to the accumbens, were associated with shorter sleep durations. This finding aligns with the observations of previous studies, which suggest that the maturation of the DMN's connectivity patterns can significantly influence sleep quality and duration in a pediatric population by potentially modulating the neurobiological mechanisms that govern sleep architecture (Cosío-Guirado et al., 2024).

Although the effect sizes reported in this study are relatively small (e.g., *β* = 0.08 for the RH default network to thalamus), we believe that these findings still provide important insights and practical value for the study of the neurobiological regulation of children's sleep duration. While the influence of individual brain connections is limited, they may have cumulative or interactive effects within the entire brain network. Such network effects could have profound impacts on children's behavioral and cognitive development. However, this also highlights a limitation of the current study, where the practical impact of individual connections is difficult to apply directly in clinical practice. Future research should utilize larger sample sizes and more comprehensive analytical methods to further explore how these structural connections function within broader brain networks and assess their specific impacts on children's health.

Our findings hold significant implications for the prevention of sleep disorders in children and for the application of neurofeedback interventions, by employing the brain structural connectome as a biomarker to assess sleep quality. However, our study is subject to several limitations. Firstly, the GWAS dataset used primarily comprises individuals of European descent, and its applicability to other populations remains to be validated, necessitating further exploratory work. Secondly, although our current MR analysis has identified potential causal relationships between brain network connectivity and children's sleep duration, these results are largely based on statistical inference and require validation through further clinical randomized controlled trials (RCTs). Thirdly, while we have employed SNPs as instrumental variables, we have not explored the specific biological functions of each SNP in detail, which limits our ability to fully elucidate the underlying mechanisms. Future studies should focus on the functional significance of these specific genetic variants and investigate how they influence both brain SC and sleep duration. Despite these limitations, our study is among the first to focus on the quality of sleep in children in relation to brain SC, providing valuable insights for early interventions in pediatric sleep quality.

### Conclusion

This two-sample Mendelian randomization analysis demonstrates a causal relationship between brain structural connectomes and sleep duration in children. Our findings advance the understanding of structural and functional connectivity patterns associated with pediatric sleep, potentially leading to more targeted and effective treatments for improving sleep health in this population.

## Data Availability

The datasets used and/or analyzed during the current study are available from the corresponding author upon reasonable request.

## Synthesis

Reviewing Editor: Selva Baltan, Oregon Health &Science University Department of Surgery Decisions are customarily a result of the Reviewing Editor and the peer reviewers coming together and discussing their recommendations until a consensus is reached. When revisions are invited, a fact-based synthesis statement explaining their decision and outlining what is needed to prepare a revision will be listed below. The following reviewer(s) agreed to reveal their identity: Rupesh Chillale.

Successful revision
